# Anaesthesiological strategies in elective craniotomy: randomized, equivalence, open trial – The *NeuroMorfeo *trial

**DOI:** 10.1186/1745-6215-10-19

**Published:** 2009-04-06

**Authors:** Giuseppe Citerio, Maria Grazia Franzosi, Roberto Latini, Serge Masson, Simona Barlera, Stefano Guzzetti, Antonio Pesenti

**Affiliations:** 1 Neuroanaesthesia and Neurointensive Care Unit, Department of Perioperative Medicine and Intensive Care, San Gerardo Hospital, Monza, Milano, Italy; 2 Department of Cardiovascular Research, Istituto di Ricerche Farmacologiche Mario Negri, Milano, Italy; 3 Department of Experimental Medicine University of Milan-Bicocca, Monza, Milano, Italy; 4 Internal Medicine II, Department of Clinical Sciences, Ospedale Luigi Sacco, Via GB Grassi 74, 20157 Milano, Italy

## Abstract

**Background:**

Many studies have attempted to determine the *"best" *anaesthetic technique for neurosurgical procedures in patients without intracranial hypertension. So far, no study comparing intravenous (IA) with volatile-based neuroanaesthesia (VA) has been able to demonstrate major outcome differences nor a superiority of one of the two strategies in patients undergoing elective supratentorial neurosurgery. Therefore, current practice varies and includes the use of either volatile or intravenous anaesthetics in addition to narcotics. Actually the choice of the anaestesiological strategy depends only on the anaesthetists' preferences or institutional policies.

This trial, named NeuroMorfeo, aims to assess the equivalence between volatile and intravenous anaesthetics for neurosurgical procedures.

**Methods/Design:**

NeuroMorfeo is a multicenter, randomized, open label, controlled trial, based on an equivalence design. Patients aged between 18 and 75 years, scheduled for elective craniotomy for supratentorial lesion without signs of intracranial hypertension, in good physical state (ASA I-III) and Glasgow Coma Scale (GCS) equal to 15, are randomly assigned to one of three anaesthesiological strategies (two VA arms, sevoflurane + fentanyl or sevoflurane + remifentanil, and one IA, propofol + remifentanil). The equivalence between intravenous and volatile-based neuroanaesthesia will be evaluated by comparing the intervals required to reach, after anaesthesia discontinuation, a modified Aldrete score ≥ 9 (primary end-point). Two statistical comparisons have been planned:

1) sevoflurane + fentanyl vs. propofol + remifentanil;

2) sevoflurane + remifentanil vs. propofol + remifentanil.

Secondary end-points include: an assessment of neurovegetative stress based on (a) measurement of urinary catecholamines and plasma and urinary cortisol and (b) estimate of sympathetic/parasympathetic balance by power spectrum analyses of electrocardiographic tracings recorded during anaesthesia; intraoperative adverse events; evaluation of surgical field; postoperative adverse events; patient's satisfaction and analysis of costs.

411 patients will be recruited in 14 Italian centers during an 18-month period.

**Discussion:**

We presented the development phase of this anaesthesiological on-going trial. The recruitment started December 4^th^, 2007 and up to 4^th^, December 2008, 314 patients have been enrolled.

## Background

Anaesthesia for neurosurgical procedures should ideally provide optimal surgical conditions while maintaining appropriate cerebral oxygen supply and stable systemic haemodynamics. Rapid emergence from anaesthesia is also desirable to allow a quick neurological examination at the end of procedure.

Over the years, several studies have demonstrated that in patient with intracranial hypertension the best strategy is based on intravenous anaesthesia for its beneficial cerebral haemodynamic effects, for its "neuroprotective" role and for its action on cerebral metabolism [1].

On the other hand no studies have been able to determine the "best" anaesthetic technique for neurosurgical procedures in patients without signs of cerebral hypertension. The choice of volatile or intravenous strategy is still actively debated [2].

Current practice seems to vary and includes the use of either volatile or intravenous anaesthetics in addition to various narcotics [3]. The most frequently administered anaesthetics during neurosurgical procedures are either propofol-opioid or sevoflurane-opioid [4]. Inhaled agents are preferred by some neuroanaesthetists because of the ease of their administration, the availability of end-tidal agent monitoring and lack of evidence of outcome compared with intravenous hypnotics. Other anaesthetists prefer, on the other hand, intravenous anaesthesia.

The drugs studied in this neuroanaesthesia trial include:

### Sevoflurane

Sevoflurane is a halogenated inhalational anaesthetic agent administered by vaporization and used in induction and maintenance of anaesthesia. Minimum Alveolar Concentration (MAC) in oxygen mixture for a 40 years old adult is 2.1%; MAC decreases with age. Sevoflurane has a direct vasodilator effect that increase cerebral blood flow (CBF) while reduce cerebral metabolic rate (CMRO_2_). CBF normalizes approximately 3 hours after the initial exposure to 1.3 MAC of anaesthetic.

### Propofol

Propofol is an intravenous sedative-hypnotic agent used during anaesthesia induction and maintenance. Intravenous injection of a therapeutic dose of propofol induces hypnosis usually within 40 seconds from the start of injection, the interval request for one arm-brain circulation. Steady-state propofol blood concentrations are generally proportional to infusion rates. Propofol has many of the properties of an ideal agent for neuroanaesthesia, with beneficial cerebral haemodynamic effects reducing CBF, favourable pharmacokinetics and high-quality recovery profile despite prolonged duration of infusion. It reduces CMRO_2 _to a similar extent as Sevoflurane [1].

Opioids that are usually associated with intravenous and volatile anaesthesia are remifentanil and fentanyl.

### Remifentanil

Remifentanil is an antagonist of opioid μ-receptor and it has a peculiar pharmacokinetic property due to its metabolism mediated by a non specific esterase with rapid onset and termination effects, independently by the duration of administration. It's commonly used as analgesic for induction and maintenance of anaesthesia as synergistic agent with other anaesthetics.

### Fentanyl

Fentanyl is an opioid analgesic. It interacts predominately with the opioid μ-receptor and exerts its principal pharmacologic effects on the central nervous system. Its primary actions of therapeutic value are analgesia and sedation. Fentanyl may increase the patient's tolerance for pain. Its analgesic activity is, most likely, due to its conversion to morphine.

Few studies compared propofol-based anaesthesia with other types of anaesthesia in neurosurgical patients. Todd [2] compared propofol/fentanyl with nitrous oxide/fentanyl and isoflurane/nitrous oxide in 121 patients undergoing craniotomy for supratentorial mass lesions. There were minor differences in adverse effects, but no significant differences in neurological outcome. Nitrous oxide/fentanyl patients had more hypotension during induction and more post-operative vomiting, but were quicker to awaken in recovery. Isoflurane/nitrous oxide patients had more tachycardia during induction, higher ICP prior to dural opening and longer emergence times. Finally, propofol/fentanyl patients had less haemodynamic instability during induction and less postoperative vomiting, but were slower than the nitrous oxide/fentanyl patients to wake-up. Recently Magni [5] evaluated emergence time and early postoperative cognitive function in patients who received either a volatile-based anaesthetic or a total intravenous anaesthetic. The authors demonstrated no differences in intraoperative brain volume, emergence time, early postoperative cognitive abilities, or the incidences of postoperative shivering, pain, or nausea and vomiting. However, both intraoperative hypertensive and hypotensive episodes were more common in patients anaesthetized with propofol and remifentanil.

Based on current evidence, carefully administered anaesthesia with any of a variety of agents will result in a similar outcome. To evaluate the "anaesthesia quality", we must therefore use surrogate combined endpoints. A score used in anaesthesia is the modified Aldrete score [6] that analyzes motility, respiration and oxygenation, cardiovascular stability and consciousness. The sum of the scores ranges between 0 and 10 and a adequate score at the emergence from anaesthesia is ≥ 9.

In some recent articles the modified Aldrete score has been applied to patients anaesthetized for a craniotomy. Balakrishnan [7] compared the effects of remifentanil versus fentanyl during surgery for intracranial space-occupying lesions. Anaesthesia maintenance doses of isoflurane, nitrous oxide, and opioid were at the anaesthesiologist's discretion for both groups. The percentage of patients with a normal recovery score (alert or arousable to quiet voice, oriented, able to follow commands, motor function unchanged from their preoperative evaluation, not agitated, and had modified Aldrete Scores of 9–10) at 10 min after surgery was significantly higher for remifentanil (45% vs. 18%; P = 0.005). By 20 min, the difference between groups was not maintained (P = 0.27).

Talke [8] compared three anaesthetic techniques (inhalation, intravenous, mixed) in patients undergoing craniotomy for supratentorial intracranial surgery. None of the recovery event times (open eyes, extubation, follow commands, oriented, Aldrete score) or psychomotor test performance differed significantly. Mean interval to obtain an Aldrete score ≥ 9 was 15 minutes in all three groups. This prospective, randomized clinical study found that the three anaesthetics did not differ in intra- or postoperative haemodynamic stability or early postoperative recovery variables.

Boztug [9] investigated the role of using the bispectral index (BIS) in recovery from anaesthesia and altering drug administration in patients undergoing craniotomy.

Times to first spontaneous breathing, eye opening, and extubation (P = 0.035, P = 0.001, and P = 0.0001, respectively) were significantly shorter in the BIS-guided group. Time to an Aldrete score of 9–10 (24 ± 6 vs. 27 ± 6 minutes) and adequate neurological assessment was similar between the groups.

Del Gaudio [10] compared the use of remifentanil and fentanyl during elective supratentorial craniotomy in a target controlled infusion (TCI)-propofol anaesthesia regimen and evaluated the quality of recovery from anaesthesia. Intervals for an Aldrete score of 9–10 were respectively about 8.6 ± 1.6 minutes and 14.6 ± 2.6 minutes.

Lauta, in a preliminary report presented at the 2003 SNACC meeting [11], demonstrated similar times to an Aldrete ≥ 9 (median 5 minutes) for both volatile and intravenous anaesthesia.

Since the impact of both anaesthesiological strategies on the outcome of neurosurgical procedures is similar, we decided to compare different endpoints such as time to reach consciousness. So far, in patients undergoing elective supratentorial neurosurgery, no study comparing intravenous with volatile-based neuroanaesthesia has been able to demonstrate neither major outcome differences nor a superiority of one of the two strategies [3,5]. Rather contrasting results has been published concerning secondary anaesthesia effects like intraoperative brain volume, early postoperative cognitive ability, incidence of postoperative shivering, pain, nausea and vomiting [8].

This paper presents the design of the NeuroMorfeo study, an open trial comparing volatile versus intravenous anaesthesia strategies in patients undergoing elective supratentorial neurosurgery.

## Methods

The NeuroMorfeo study is a multicenter randomized, open label, controlled trial, with equivalence design [12], evaluating volatile anaesthesia vs. intravenous anaesthesia strategies in patients undergoing supratentorial elective neurosurgery. Enrolment criteria, evaluated in all patients during a routine preoperative assessment and physical examination, are summarized in the appendix.

Before anaesthesia induction, patient is premedicated with Midazolam (5 mg IV). An isotonic crystalloid saline solution (7–10 mL/kg) is infused through a peripheral intravenous catheter and a second line is inserted for drug administration. All patients are preoxygenated for 3 minutes with a reservoir bag in 100% O_2_.

In all patients, anaesthesia is induced with:

- Propofol (2–3 mg/kg IV),

- Fentanyl (2 to 4 μg/kg IV) in the group 1 and remifentanil (0.25 μg/kg/min IV infused for 3 minutes before induction) in groups 2 and 3.

- Cisatracurium (0.1–0.2 mg/kg IV).

After intubation of the trachea, patients are mechanically ventilated with an inspired mixture of air and oxygen (2:1). Ventilation, using a closed breathing system (fresh gas flow of 0.75 L/min oxygen and 1.5 L/min air during anaesthesia), is adjusted to achieve an end-tidal carbon dioxide of 30–35 mmHg. No local anaesthesia is allowed.

Therefore anaesthesia is maintained according to one of these three different study groups:

1. (IF) ***sevoflurane + fentanyl***: sevoflurane is maintained in a 0.75 to 1.25 MAC range and fentanyl (2–3 μg/kg/hr or 0.7 μg/kg boluses). Just before incision of the scalp, fentanyl (1–2 μg/kg/hr) can be supplemented, if necessary;

2. (IR) ***sevoflurane + remifentanil***: sevoflurane is maintained in a 0.75 to 1.25 MAC range and remifentanil (0.05–0.25 μg/kg/min reduced to 0.05–0.1 μg/kg/min after dural opening). Just before incision of the scalp, remifentanil can be supplemented, if necessary;

3. (ER) ***propofol + remifentanil***: propofol is maintained with continuous infusion at 10 mg/kg/h for the first 10 minutes, then reduced to 8 mg/kg/h for the following 10 minutes and reduced to 6 mg/kg/h thereafter and remifentanil 0.05–0.25 μg/kg/min reduced to 0.05–0.1 μg/kg/min after dural opening. Just before incision of the scalp, remifentanil could be supplemented, if necessary.

During surgery all patients are paralyzed with cisatracurium (0.1 mg/kg/h), stopped once the bone flap is secured.

At the end of surgery, residual neuromuscular blockade will be antagonized with neostigmine 2.5 mg and atropine 1 mg.

Sevoflurane and propofol infusions are reduced once the bone flap is secured and stopped at skin dressing. Fentanyl is stopped at skin dressing and remifentanil reduced at skin dressing by 30% every 3–4 minutes.

Analgesia is started before bone flap repositioning with paracetamol and morphine 0.03–0.1 mg/kg IV in remifentanil groups.

Patients are randomly assigned to one of these three strategies with equal probability. Balanced randomization is maintained at each clinical site using a stratified randomization scheme. Patients are randomized the day before surgery, once the patient has provided written informed consent and satisfied all the study eligibility criteria. The patient identification and treatment allocation are provided by the central randomization service through an interactive voice response system (IVRS). After randomization a confirmation e-mail with randomization details is automatically sent to the center. In order to minimize the possibility of bias in reporting and assessing primary and secondary endpoints, the trial adopted a PROBE design (Prospective Randomized Open trial with Blinded Evaluation of outcomes).

The primary end point is the post-anaesthesia recovery, assessed as the interval required to reach an Aldrete score ≥ 9 [6]. The Aldrete Recovery Score, which sets the standards for post-anaesthesia discharge criteria for patients, is a score (range 0–10, Table 1) used by doctors and nurses in the operating rooms. Interval (minutes; seconds) required from patient extubation to reach a modified Aldrete score ≥ 9 is evaluated in each patient.

**Table 1:** The Aldrete Score

**ACTIVITY**	2 = Able to move spontaneously or on command 4 extremities
	1 = Able to move voluntarily or on command 2 extremities
	0 = Unable to move any extremities
**RESPIRATION**	2 = Able to deep breath and cough freely
	1 = Dyspnea, shallow or limited breathing
	0 = Apneic

**CIRCULATION**	2 = BP + 20 mmHg of pre-sedation level
	1 = BP + 20–50 mmHg of pre-sedation level
	0 = BP + 50 mmHg of pre-sedation level

**CONSCIOUSNESS**	2 = Fully awake
	1 = Arousable on calling
	0 = Not responding

**SKIN COLOR**	2 = Normal
	1 = Pale, dusky, blotchy, jaundiced, other
	0 = Cyanotic

The following comparisons are planned:

- *Sevoflurane + remifentanil *versus *propofol + remifentanil*;

- *Sevoflurane + fentanyl *versus *propofol + remifentanil *[7,10].

The Aldrete score is assessed by a trained anaesthesiologist, blinded to allocation treatment group. Every 30–60 seconds the anaesthesiologist checks the patients' activity (ability to move extremities), respiration (ability to cough and breath), circulation (level of blood pressure compared to patient personal baseline), consciousness (ability to keep himself awake), colour (level of peripheral oxygen saturation).

Every anaesthesiologist involved in the evaluation of the score has been trained and certified with a dedicated software course developed to use the Aldrete score.

**Figure 1:**
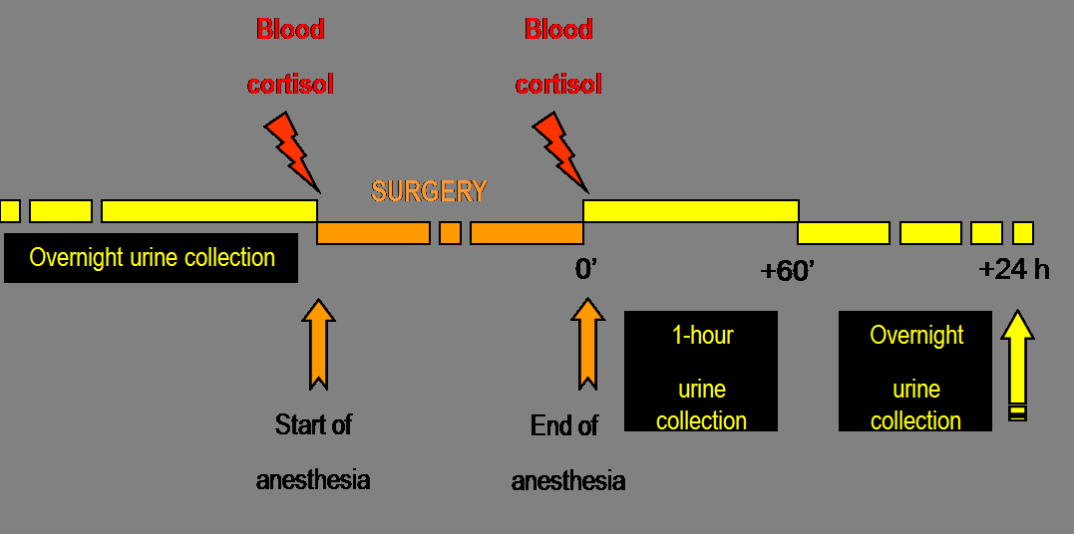
**Scheme of blood and urine samples for stress biomarkers (cortisol and catecholamines)**. For each patient samples are collected before the induction, during surgical procedure and after awakening to evaluate within-patient changes.

*Secondary end points *are:

1. Anaesthesia-related neurovegetative stress evaluation through the measurement of:

a) Haemodynamic stability.

b) Stress biomarkers (cortisol and catecholamines) [13]. Blood and urine samples are collected before the induction, during surgical procedure and after awakening to evaluate within-patient changes in biomarkers (as shown in figure 1). [14-18]

c) Cardiac autonomic function (dynamic analysis of the ECG)[19,20] Cardiac autonomic function tracings and biomarkers are assessed in central facilities by expert personnel blinded to the assigned treatment.

2. Intraoperative adverse events assessment: arterial hypotension and hypertension, bradycardia and tachycardia, osmotics and hyperventilation requirements [21].

3. Brain relaxation is assessed at dural opening, by the neurosurgeon, blinded to the study group, using a 4-point brain relaxation score [22,23]:

a) Relaxed brain.

b) Mild brain swelling, acceptable.

c) Moderate brain swelling, no therapy required.

d) Severe swelling, requiring treatment.

4. Post-operative adverse events assessment as seizures, cough, shivering, agitation, cerebral haematoma and post-operative pain.

5. Evaluation of patient's satisfaction through the filling of the *The Iowa Satisfaction with Anaesthesia Scale (ISAS*, table 2[24]) 24 hours after surgery.

**Table 2:** The Iowa Satisfaction with Anaesthesia Scale (ISAS)

I threw up or felt like throwing up	*Disagree very much*
	*Disagree moderately*
	*Disagree slightly*
	*Agree slightly*
	*Agree moderately*
	*Agree very much*
I would want to have the same anaesthetic again	*Disagree very much*
	*Disagree moderately*
	*Disagree slightly*
	*Agree slightly*
	*Agree moderately*
	*Agree very much*

I itched	*Disagree very much*
	*Disagree moderately*
	*Disagree slightly*
	*Agree slightly*
	*Agree moderately*
	*Agree very much*

I felt relaxed	*Disagree very much*
	*Disagree moderately*
	*Disagree slightly*
	*Agree slightly*
	*Agree moderately*
	*Agree very much*

I felt pain	*Disagree very much*
	*Disagree moderately*
	*Disagree slightly*
	*Agree slightly*
	*Agree moderately*
	*Agree very much*

I felt safe	*Disagree very much*
	*Disagree moderately*
	*Disagree slightly*
	*Agree slightly*
	*Agree moderately*
	*Agree very much*

I was too cold or hot	*Disagree very much*
	*Disagree moderately*
	*Disagree slightly*
	*Agree slightly*
	*Agree moderately*
	*Agree very much*

I was satisfied with my anaesthetic care	*Disagree very much*
	*Disagree moderately*
	*Disagree slightly*
	*Agree slightly*
	*Agree moderately*
	*Agree very much*

I felt pain during surgery	*Disagree very much*
	*Disagree moderately*
	*Disagree slightly*
	*Agree slightly*
	*Agree moderately*
	*Agree very much*

I felt good	*Disagree very much*
	*Disagree moderately*
	*Disagree slightly*
	*Agree slightly*
	*Agree moderately*
	*Agree very much*

I hurt	*Disagree very much*
	*Disagree moderately*
	*Disagree slightly*
	*Agree slightly*
	*Agree moderately*
	*Agree very much*

6. Evaluation of the costs of the three strategies.

Baseline characteristics of the patient (including demographics, medical history, physical exam, vital signs and serial lab tests, ASA, Body Mass Index (BMI), planned intracranial surgery) and all the variables registered during and after the neurosurgery as haemodynamic parameters (arterial pressure and heart rate), diuresis, body temperature, arterial saturation, blood gas analysis, end-tidal concentrations of anaesthetic vapour, oxygen, and carbon dioxide, intraoperative and post-operative adverse events, are recorded on a Case Report Form (CRF). Data collection ends 24 hours after the end of surgery.

A CRF, software based, has been develop and distributed to the centers. Data are inputted at each centre and are sent in an encrypted format to the coordinating centre for storage in a central database and for statistical analysis. A monitoring program, according to GCP rules, has been planned. It includes a central monitoring activity for efficacy and safety and an on-site monitoring. Central and on site monitoring activity are carried out by Mario Negri Institute's experienced personnel and includes CRFs reviewing in term of completeness and accuracy, errors and omissions. All corrections are entered on data query forms that are sent to the Investigator. On-site monitoring consists in at least four visits for each participating site: a) an initiation visit before starting the recruitment, b) a visit after the third randomized patient, c) a visit after 15 recruited patients and d) a close-out visit. During this visit the clinical monitor reviews on site all CRF and written informed consents. Accuracy of the key data is verified reviewing the source documents filed at the Investigator's site and the clinical records.

An International Data and Safety Monitoring Board (DSMB) guarantee the patients' safety during the study. Serious adverse events (SAEs) are collected on the CRF and evaluated by the DSMB. Investigators are required to report to the Coordinating Centre all the SAEs suspected to be related to the study medications within 24 hours from their occurrence.

Fourteen Italian neuroanaesthesia departments have agreed to participate to the study. These centers have been selected on the basis of participation to previous multicenter studies coordinated by San Gerardo Neurointensive Care, Monza. These multicenter studies are Neurolink [25], a survey on head injury in 28 Italian Neurosurgical Hospitals (1997), Neurolink Domestic (on more than 1600 severely head injured patients, recruited from 1997 – still ongoing), BrainIT [26] (coordination of the Italian centers, project supported by the EU framework V (EEC Project:QLG3-2002-01160) ), ESAnet (data collection in subarachnoid haemorrhage patients in 23 Neurosurgical Departments [27]).

A web blog  has been developed for facilitating the communication between the participating centers.

### Statistical Analysis

This trial has the objective to evaluate if IR, as well as IF, is equivalent to ER. As described previously, this objective will be addressed by evaluation of the interval required to reach an Aldrete score ≥ 9. To test for equivalence two comparisons are planned:

1. IR vs. ER,

2. IF vs. ER.

The difference between the groups has been estimated on a clinical judgment basis.

The estimate for the mean value and the standard deviation of each group integrates also information from the limited published literature.

Having a mean duration of the neurosurgical procedures in the enrolling centers (and this information is available because we did monitor > 100 neurosurgical procedures in the 14 centers) of >300 minutes, for both the comparisons, it has been estimated that plausible equivalence limits for the mean difference in the time to reach an Aldrete score ≥ 9 range from ± 3 minutes with pooled standard deviation equal to 7.

This evaluation comes from:

• The need of a rapid emergence from anaesthesia to allow a quick neurological examination at the end of procedure. Nevertheless this emergence is not instantaneous because prolonged administration of anesthesiological drugs (> 300 minutes) and their pharmacokinetic properties requires variable interval from their discontinuation to obtain an Aldrete score ≥ 9. This score considers many items as motor activity, adequate respiration, normal circulation and peripheral perfusion and recover of consciousness. For reaching the maximum values (9–10) the patient have to be completely awake, with normal circulation and respiratory, and be cooperative. On a pure clinical judgment, ± 180 seconds, after > 300 minutes of anaesthesia are a very short period. Based on a pure clinical judgment this interval is extremely reasonable.

• The evaluation that this value is less than 1% of the total surgical duration.

We selected an equivalence design because these differences aren't clinically significant and in our opinion the strategies, according the primary endpoint, are similar. Differences between the strategies will be evaluated analyzing multiple secondary endpoints.

The study assumes a 12–18 months of patients' enrolment, a 10% drop-out rate and a overall significance level ≤ 0.05, taking into account that each comparison will be tested at the significance level = 0.025.

A study sample size of 411 patients (137 in each group) is estimated, since it will provide at least a power ≥ 84% to reject the equivalence hypothesis. Sample size calculation has been performed applying a two-group t-test of equivalence in means performed with the statistical package Nquery Advisor 6.01.

The intention to treat (ITT) population, consisting of all randomized patients originally allocated to therapy specific anaesthesia arm at the time of randomization will be used for the efficacy analysis. In addition a per protocol (PP) analysis will be performed on the efficacy endpoint. If the results of the two approaches will be somehow controversial, the PP analysis will be regarded as the definitive one being in the context of an equivalence trial. The primary efficacy outcome of the study is the time to reach an Aldrete score ≥ 9. The conclusion that IR as well as IF are equivalent to ER will be drawn if the lower limit of the one-sided 95% interval around the mean difference is greater than -3 minutes and the upper limit of the one-sided 95% confidence interval around the mean difference is lower than 3 minutes. [28-30] Differences between the two randomized groups will be analyzed by means of analysis of variance or by a non-parametric approach if distributional assumptions are not satisfied. Any clinically significant imbalance between the randomized groups will be considered for use as covariates. Secondary and other efficacy outcomes of the study will be analyzed using the same statistical methodology adopted for the primary efficacy outcome. Safety analysis will be carried out using the ITT population to allow a benefit/risk assessment within the same study population.

## Results

The first patient has been recruited on December 4, 2007. In the following 6 months, all the centers have obtained local IRB approval and the initiation visit. To date (4^th^, December 2008) 314 patients have been enrolled in the study. The enrolment should be completed during the second quarter of 2009.

## Discussion

NeuroMorfeo trial has been designed and initiated in the context of an absence of evidence about the "best" anaesthetic strategy for supratentorial elective neurosurgical procedures. Several studies have compared intravenous with volatile-based neuroanaesthesia without conclusive results in favour of one of the two strategies. Clinical research is usually based on the concept of developing new therapeutic strategies able to demonstrate a better efficacy compared with those available, testing a null hypothesis. Under certain circumstances, however, it may be inappropriate to plan a trial around a null hypothesis. The NeuroMorfeo study is a model case. Knowing that several studies have tried to demonstrate a superiority of one anaesthesiological strategy without conclusive results, we decided to plan a study based on an equivalence design and that is the innovative part of this protocol. Some conditions have to be satisfied before choosing an equivalence design. First, the treatment under consideration exhibits therapeutic non-inferiority to the active control, that means in our field that no evidence about the best anaesthesiological practice in elective neurosurgery is available. Moreover, the tested treatment could offer ancillary, even important, advantages in safety, tolerability, cost, or convenience. These all are the relevant aspects that this study is going to investigate.

An ideal neuroanesthesia should maintain an appropriate cerebral oxygen supply and stable systemic haemodynamic as well as rapid emergence time to allow a quick neurological examination at the end of procedure.

This is the main reason for choosing the Aldrete score as first, simple, measurable, endpoint. The Aldrete score is the principal score used in literature to evaluate the post anaesthesia awakening.

Secondary end points have no less importance than the first one. This research will allow exploring many aspects of modern neuroanaesthesia, as secondary endpoints. In fact, we will compare these strategies in terms of neurovegetative activation (haemodynamic stability, essay of biomarkers of stress, cardiac autonomic function), intraoperative and post-operative adverse events, state of brain relaxation, patient's satisfaction and costs of the three strategies.

To minimize bias in assessing the treatment effects, we adopted a PROBE design: all the evaluation of haemodynamic stability, biomarkers of stress, cardiac autonomic function, surgical field and post-operative adverse events are done by personnel blinded to the assigned treatment.

The design and formulation of this protocol will enable us to reach a conclusion about the "best" elective neurosurgical anaesthesiological strategy.

## Competing interests

The authors declare that they have no competing interests.

## Authors' contributions

GC, principal investigator, is responsible for coordinating the NeuroMorfeo study. All authors contributed to the design of the study and to draft the manuscript, and approved the final version. SB is responsible for statistics and data analysis. All authors will participate in interpretation of results.

## Appendix

### Inclusion and exclusion criteria

#### Inclusion criteria

- patient scheduled for elective intracranial surgery under general anaesthesia for a supratentorial mass lesion;

- physical state, evaluated with the ASA (American Society of Anaesthesiologists, ) classification I (normal healthy patient), II (patient with mild systemic disease), or III (patient with severe systemic disease);

- age 18–75 years;

- normal preoperative level of consciousness, i.e. Glasgow Coma Scale (GCS) equal to 15;

- no clinical signs of intracranial hypertension.

#### Exclusion criteria

- Severe cardiovascular pathology, as uncontrolled arterial hypertension and documented reduced coronary reserve;

- Renal or liver disease precluding the use of either anaesthetic technique;

- Pregnancy;

- Known allergies to any anaesthetic agent;

- Reduced preoperative level of consciousness, i.e. Glasgow Coma Scale (GCS) < 15;

- Body weight greater than 120 kg;

- History of drug abuse or psychiatric conditions;

- Documented disturbance of the hypothalamic region;

- Refusal to sign consent form;

- Participation in other clinical trials in the last 2 months;

- Planned awakening in ICU, due to the location and/or size of the lesion, postoperative sedation and postoperative mechanical ventilation requirements.
